# Pooled analysis of drug-related interstitial lung disease and/or pneumonitis in nine trastuzumab deruxtecan monotherapy studies☆

**DOI:** 10.1016/j.esmoop.2022.100554

**Published:** 2022-08-11

**Authors:** C.A. Powell, S. Modi, H. Iwata, S. Takahashi, E.F. Smit, S. Siena, D.-Y. Chang, E. Macpherson, A. Qin, J. Singh, C. Taitt, N. Shire, D. Ross Camidge

**Affiliations:** 1 Catherine and Henry J. Gaisman Division of Pulmonary Critical Care and Sleep Medicine, Icahn School of Medicine at Mount Sinai, New York, USA; 2 Memorial Sloan Kettering Cancer Center, New York, USA; 3 Aichi Cancer Center Hospital, Nagoya, Aichi, Japan; 4 Medical Oncology, The Cancer Institute Hospital of JFCR, Koto, Tokyo, Japan; 5 Department of Thoracic Oncology, Netherlands Cancer Institute, Amsterdam, The Netherlands; 6 Department of Oncology and Hemato-Oncology, Università degli Studi di Milano, Milan; 7 Niguarda Cancer Center, Grande Ospedale Metropolitano Niguarda, Milan, Italy; 8 National Taiwan University Hospital, Taipei City, Taiwan; 9 AstraZeneca Pharmaceuticals, Gaithersburg, USA; 10 Daiichi Sankyo Inc., Basking Ridge, USA; 11 University of Colorado Cancer Center, Aurora, USA

**Keywords:** trastuzumab deruxtecan, adverse event, interstitial lung disease, pneumonitis, HER2

## Abstract

**Introduction:**

This pooled analysis of nine phase I and II trastuzumab deruxtecan (T-DXd) monotherapy studies described drug-related interstitial lung disease (ILD)/pneumonitis in patients treated with T-DXd.

**Methods:**

Patients who received T-DXd across nine studies were included. Investigator-assessed ILD/pneumonitis events were retrospectively reviewed by an independent adjudication committee; events adjudicated as drug-related ILD/pneumonitis are summarized.

**Results:**

The analysis included 1150 patients (breast cancer, 44.3%; gastric cancer, 25.6%; lung cancer, 17.7%; colorectal cancer, 9.3%; other cancer, 3.0%). Median treatment duration was 5.8 (range, 0.7-56.3) months, with a median of 4 (range, 1-27) prior lines of therapy. The overall incidence of adjudicated drug-related ILD/pneumonitis was 15.4% (grade 5, 2.2%). Most patients with ILD/pneumonitis experienced low-grade events (grade 1 or 2, 77.4%); 87.0% had their first event within 12 months [median, 5.4 (range, <0.1-46.8) months] of their first dose of T-DXd. Based on data review, adjudicated ILD/pneumonitis onset occurred earlier than identified by investigators for 53.2% of events [median difference in onset date, 43 (range, 1-499) days]. Stepwise Cox regression identified several baseline factors potentially associated with increased risk of adjudicated drug-related ILD/pneumonitis: age <65 years, enrollment in Japan, T-DXd dose >6.4 mg/kg, oxygen saturation <95%, moderate/severe renal impairment, presence of lung comorbidities, and time since initial diagnosis >4 years.

**Conclusions:**

In this pooled analysis of heavily treated patients, the incidence of ILD/pneumonitis was 15.4%, with most being low grade and occurring in the first 12 months of treatment. The benefit–risk of T-DXd treatment is positive; however, some patients may be at increased risk of developing ILD/pneumonitis, and further investigation is needed to confirm ILD/pneumonitis risk factors. Close monitoring and proactive management of ILD/pneumonitis are warranted for all.

## Highlights

• Interstitial lung disease (ILD)/pneumonitis is a significant adverse event related to trastuzumab deruxtecan (T-DXd).
• This pooled analysis of nine T-DXd monotherapy studies evaluated ILD/pneumonitis risk in 1150 heavily pretreated patients.
• Overall incidence of adjudicated T-DXd-related ILD/pneumonitis was 15.4% (grade 1 or 2, 77.4%; grade 5, 2.2%).
• Within 12 months of their first T-DXd dose, 87.0% of patients had their first event [median, 5.4 (range, <0.1-46.8) months].
• Proactive monitoring and prompt diagnosis and management are important to improving ILD/pneumonitis event outcomes.

## Introduction

Trastuzumab deruxtecan (T-DXd) is an antibody–drug conjugate (ADC) composed of an anti-human epidermal growth factor receptor 2 (HER2) humanized monoclonal antibody, a tetrapeptide-based cleavable linker, and a topoisomerase I inhibitor payload, which is present at a high drug-to-antibody ratio (∼8:1).^1^^,^^2^ The linker is stable in plasma but selectively cleaved by cathepsins, which may be up-regulated in cancer cells.^2^, ^3^, ^4^, ^5^ The released payload is membrane permeable, allowing for an antitumor bystander effect, but has a short half-life, potentially minimizing systemic exposure.^2^^,^^4^

T-DXd is being investigated in a series of trials (DESTINY) in several indications. Data from the phase II DESTINY-Breast01 (NCT03248492; DS8201-A-U201) and DESTINY-Gastric01 (NCT03329690; DS8201-A-J202) studies led to approvals of T-DXd in multiple countries for HER2-positive unresectable or metastatic breast cancer that progressed on two or more prior therapies (accelerated approval in the USA) and HER2-positive locally advanced or metastatic gastric cancer.^6^, ^7^, ^8^, ^9^, ^10^, ^11^, ^12^ Recently, T-DXd has been approved in the USA as a second-line treatment of patients with HER2-positive unresectable or metastatic breast cancer based on data from the randomized phase III DESTINY-Breast03 (NCT03529110; DS8201-A-U302) study of T-DXd versus trastuzumab emtansine (T-DM1) in patients with HER2-positive metastatic breast cancer.^7^^,^^13^ Clinical investigation is ongoing in several other populations.^14^, ^15^, ^16^, ^17^, ^18^

Interstitial lung disease (ILD)/pneumonitis is a known risk with a variety of cancer therapies, including multiple ADCs.^19^, ^20^, ^21^, ^22^, ^23^ T-DXd has demonstrated a generally manageable safety profile across the DESTINY clinical program, with hematologic and gastrointestinal adverse events (AEs) being the most common; ILD/pneumonitis has been identified as an AE of special interest.^1^^,^^6^, ^7^, ^8^, ^9^, ^10^, ^11^, ^12^^,^^14^^,^^18^^,^^24^, ^25^, ^26^, ^27^, ^28^, ^29^, ^30^, ^31^ The mechanism of T-DXd-related ILD/pneumonitis has not been fully delineated. A recent study in cynomolgus monkeys suggested that alveolar macrophage uptake and redistribution of T-DXd could be involved.^32^

Study-specific guidelines for monitoring, evaluating, and managing ILD/pneumonitis in T-DXd clinical trials, including dose modification and supportive care recommendations, were updated in 2019 (Table 1) with more specific details regarding dosages and duration of steroid treatment, including for grade 1 events, than previous guidelines.^11^^,^^14^^,^^33^ The approved labels for T-DXd also include warnings regarding ILD/pneumonitis and guidelines for its management.^6^, ^7^, ^8^, ^9^, ^10^

**Table 1:** Recommended guidelines for management of T-DXd–induced interstitial lung disease (updated in 2019)^14^

	Grade 1	Grade 2	Grade 3 or 4
Work-up	• If a patient develops radiographic changes potentially consistent with ILD/pneumonitis or develops an acute onset of new or worsening pulmonary or other related signs/symptoms such as dyspnea, cough, or fever, rule out ILD/pneumonitis • If the AE is confirmed to have an etiology other than ILD/pneumonitis, follow routine clinical practice • Evaluations should include: ○ High-resolution CT ○ Pulmonologist consultation (infectious disease consultation as clinically indicated) ○ Blood culture and CBC. Other blood tests could be considered as needed ○ Consider bronchoscopy and bronchoalveolar lavage if clinically indicated and feasible ○ Pulmonary function tests and pulse oximetry (SpO_2_) ○ Arterial blood gases if clinically indicated ○ One blood sample collection for PK analysis as soon as ILD/pneumonitis is suspected, if feasible ○ Other tests could be considered, as needed • If the AE is confirmed to be ILD/pneumonitis, follow the ILD/pneumonitis management guidance as outlined below • All events of ILD/pneumonitis regardless of severity or seriousness will be followed until resolution, including after drug discontinuation		
Dose modification	• The administration of T-DXd must be interrupted • T-DXd can be restarted only if the event is fully resolved to grade 0: ○ If resolved in ≤28 days from day of onset, maintain dose ○ If resolved in >28 days from day ofonset, reduce dose 1 level • However, if the grade 1 ILD/pneumonitis occurs beyond day 22 and has not resolved within 49 days from the last infusion, the drug should be discontinued	Permanently discontinue patient from T-DXd treatment	Permanently discontinue patient from T-DXd treatment
Toxicity management	• Monitor and closely follow up in 2-7 days for onset of clinical symptoms and pulse oximetry • Consider follow-up imaging in 1-2 weeks (or as clinically indicated) • Consider starting systemic steroids (e.g. ≥0.5 mg/kg/day of prednisone or equivalent) until improvement, followed by gradual taper over ≥4 weeks • If diagnostic observations worsen despite initiation of corticosteroids, follow grade 2 guidelines. (If the patient is asymptomatic, the toxicity should still be considered as grade 1 even if glucocorticoids were given)	• Promptly start systemic glucocorticoids (e.g. ≥1 mg/kg/day of prednisone or equivalent) for ≥14 days or until complete resolution of clinical and chest CT findings, then followed by gradual taper over ≥4 weeks • Monitor symptoms closely • Re-image as clinically indicated • If clinical or diagnostic observations worsen or do not improve in 5 days: ○ Consider increasing dose of steroids (e.g. 2 mg/kg/day of prednisone or equivalent), switching administration to i.v. (e.g. methylprednisolone) ○ Reconsider additional work-up for alternative etiologies as described above ○ Escalate care as clinically indicated	• Hospitalization required • Promptly initiate empirical high-dose methylprednisolone i.v. treatment (e.g. 500-1000 mg/day for 3 days), followed by ≥1 mg/kg/day of prednisone (or equivalent) for ≥14 days or until complete resolution of clinical and chest CT findings, then followed by gradual taper over ≥4 weeks • Re-image as clinically indicated • If still no improvement within 3-5 days: ○ Reconsider additional work-up for alternative etiologies as described above ○ Consider other immunosuppressants and/or treat per local practice

AE, adverse event; CBC, complete blood count; CT, computed tomography; ILD, interstitial lung disease; i.v., intravenous; PK, pharmacokinetics; q3w, every 3 weeks; T-DXd, trastuzumab deruxtecan.

From *New England Journal of Medicine*, Li BT, Smit ET, Goto Y, et al. Trastuzumab deruxtecan in *HER2*-mutant non–small-cell lung cancer. Volume 386, Issue 3, Pages 241-251. Copyright © 2021 Massachusetts Medical Society. Reprinted with permission from Massachusetts Medical Society.

To characterize T-DXd-related ILD/pneumonitis, we conducted a pooled analysis of nine phase I and II T-DXd monotherapy studies across multiple tumor types: DS8201-A-J101 (NCT02564900), DS8201-A-J102 (NCT03366428), DS8201-A-A103 (NCT03368196), DS8201-A-A104 (NCT03383692), DESTINY-Breast01, DESTINY-Gastric01, DESTINY-CRC01 (NCT03384940; DS8201-A-J203), DESTINY-Lung01 (NCT03505710; DS8201-A-U204), and DESTINY-Gastric02 (NCT04014075; DS8201-A-U205). This pooled analysis includes data from both before and after the updated guidelines were published and provides a snapshot of the current diagnosis and management of ILD/pneumonitis in T-DXd trials.

## Materials and methods

### Study populations

**Figure 1:**
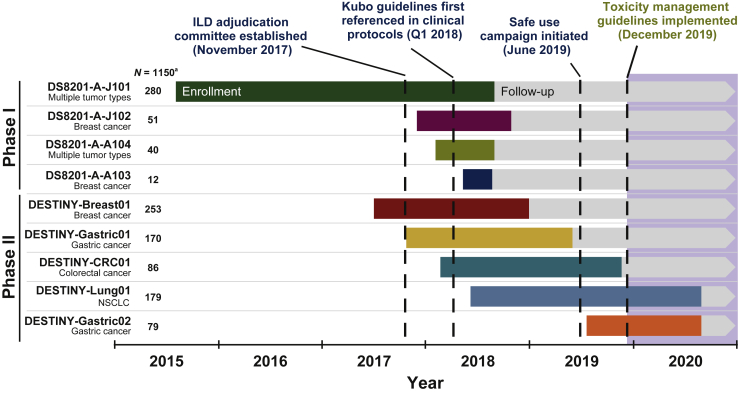
**Studies and patients included in the pooled analysis.** The colored bar on each arrow indicates the time of patient enrollment and the gray is follow-up.^1^^,^^11^^,^^12^^,^^14^^,^^26^^,^^30^^,^^31^^,^^33^^,^^34^^,^^43^ All studies noted here are active but no longer recruiting, except for DESTINY-Gastric01 and DESTINY-CRC01, which were completed in 2020.^12^^,^^30^ Note that most patients were enrolled before the implementation of toxicity management guidelines. ILD, interstitial lung disease; NSCLC, non-small-cell lung cancer; Q, quarter. ^a^Only patients who received trastuzumab deruxtecan 5.4, 6.4, 7.4, or 8.0 mg/kg every 3 weeks are included.

Data for these analyses were derived from four phase I and five phase II T-DXd monotherapy studies, which are detailed in Figure 1 and Supplementary Table S1, available at https://doi.org/10.1016/j.esmoop.2022.100554. T-DXd doses varied across studies; these analyses included data from patients who received T-DXd 5.4, 6.4, 7.4, or 8.0 mg/kg every 3 weeks (q3w).

### Adjudication committee

An independent adjudication committee (AC) was established in November 2017 after the first fatal ILD/pneumonitis event suspected to be T-DXd related was reported by an investigator during a clinical trial.^34^ The AC retrospectively reviews all potential ILD/pneumonitis cases [i.e. any AE reported under a defined set of preferred terms (Supplementary material, available at https://doi.org/10.1016/j.esmoop.2022.100554)] using imaging [including all available chest computed tomography (CT) images related to the potential ILD/pneumonitis case, such as those acquired at baseline before administration of study drug and subsequent chest CT imaging studies] and clinical data (from baseline to the time of the potential ILD/pneumonitis case) to independently assess whether the reported event was ILD/pneumonitis and, if so, whether it was related to T-DXd. All ILD/pneumonitis events adjudicated as T-DXd related were analyzed for this report.

The AC assessed the grade and date of onset of each event. Grading of ILD/pneumonitis events was based on criteria for grading of pneumonitis in the National Cancer Institute Common Terminology Criteria for Adverse Events (CTCAE) version 5.0 (Supplementary material, available at https://doi.org/10.1016/j.esmoop.2022.100554).^35^ In most cases where steroids were administered for asymptomatic events, the ILD/pneumonitis event was adjudicated as grade 2 based on steroid use per CTCAE grading criteria. For cases that were adjudicated after the issuance of current guidelines, all asymptomatic events were considered to be grade 1, regardless of steroid use, consistent with those recommendations (as detailed in the Supplementary material, available at https://doi.org/10.1016/j.esmoop.2022.100554). In addition, patients who died and had an ongoing ILD/pneumonitis event assessed by the AC as related to the study drug at the time of death were assessed to determine whether death was due to ILD/pneumonitis.

### Statistical analysis

Time of exposure to T-DXd and adjudicated drug-related ILD/pneumonitis by tumor type and grade were summarized. Patients with multiple adjudicated drug-related ILD/pneumonitis events were included in the summary statistics based on the event with the worst grade.

A Kaplan–Meier analysis of the time to the first adjudicated drug-related ILD/pneumonitis event, defined as the time from the first T-DXd dose until adjudicated ILD/pneumonitis onset, was carried out. Treatment discontinuations due to reasons other than ILD/pneumonitis were included as competing events for those who did not have an ILD/pneumonitis event.

Investigator-assessed versus AC-assessed onset dates were compared. For patients who had multiple ILD/pneumonitis events as reported by the investigator that were identified by the AC as a continuous event, the earliest onset reported by the investigator was used for the comparison of onset dates.

The analysis of corticosteroid use in patients with adjudicated drug-related ILD/pneumonitis considered any corticosteroids, regardless of the type, dose, number of doses, or reason for administration, initiated within 90 days of the adjudicated ILD/pneumonitis onset date.

A multivariate stepwise Cox regression model, stratified by tumor type (breast versus non-breast cancer), was used to explore the association between baseline factors and the time to adjudicated drug-related ILD/pneumonitis, with a stepwise variable selection entry criterion of *P* < 0.05 and remain criterion of *P* < 0.10. Baseline factors included in the model were age group, sex, country, Eastern Cooperative Oncology Group performance status, weight, presence of lung cancer or lung metastases/lymphangitis carcinomatosis, prior chest/lung radiotherapy, presence of lung comorbidity (defined as asthma, chronic obstructive pulmonary disease, prior ILD/pneumonitis, pulmonary fibrosis, pulmonary emphysema, and radiation pneumonitis), renal function, white blood cell count, albumin category, lines of therapy in locally advanced/metastatic setting, time since disease diagnosis category, time from end date of last anticancer therapy to first infusion of T-DXd category, dose category, and oxygen saturation (SpO_2_) category. The categorical cut-offs for potential risk factors were based on medical judgment and practical reasons (e.g. the cut-off of 95% for SpO_2_ was selected based on an exploratory analysis of a smaller dataset). This analysis was exploratory and hypothesis generating in nature (see Supplementary material, available at https://doi.org/10.1016/j.esmoop.2022.100554 for more information).

A *post hoc* exploratory analysis was conducted using the Chronic Kidney Disease Epidemiology Collaboration (CKD-EPI) formula, as recommended by the National Kidney Foundation’s Kidney Disease Outcomes Quality Initiative,^36^ to estimate glomerular filtration rate as the method for determining renal function at baseline, instead of the Cockcroft–Gault formula (see Supplementary material, available at https://doi.org/10.1016/j.esmoop.2022.100554 for more information).^37^

### Ethics

All included clinical trials were carried out in accordance with the Declaration of Helsinki and the International Conference on Harmonisation Guidelines for Good Clinical Practice. All patients provided written informed consent before enrollment.

### Data sharing statement

Anonymized individual participant data (IPD) and applicable supporting clinical study documents may be available upon request at https://vivli.org/. In cases where clinical study data and supporting documents are provided pursuant to our company policies and procedures, Daiichi Sankyo Companies will continue to protect the privacy of company and our clinical study subjects. Details on data sharing criteria and the procedure for requesting access can be found at this web address: https://vivli.org/ourmember/daiichi-sankyo/.

## Results

### Patients and baseline characteristics

Data as of 21 December 2020 were pooled from 1150 patients who received one or more dose of ≥5.4 mg/kg T-DXd monotherapy. Median age was 60.0 years (range, 20-96 years); additional baseline characteristics are summarized in Table 2. The most common tumor type was breast cancer [44.3% (510/1150)], followed by gastric cancer [25.6% (294/1150)] (Table 2). Patients had a median of 4 prior treatment regimens (range, 1-27). Most patients [93.9% (1080/1150)] had baseline SpO_2_ ≥95%.

**Table 2:** Baseline characteristics and T-DXd treatment

	*N* = 1150
Age, median (range), years ≥65 years, *n* (%)	60.0 (20-96) 396 (34.4)
Female, *n* (%)	755 (65.7)
Country, *n* (%)	
Japan	506 (44.0)
Non-Japan	644 (56.0)
ECOG PS, *n* (%)	
0	583 (50.7)
1/2	565 (49.1)/2 (0.2)
Tumor type, *n* (%)^a^	
Breast cancer	510 (44.3)
Gastric cancer	294 (25.6)
Lung cancer	203 (17.7)
Colorectal cancer	107 (9.3)
Other cancer	34 (3.0)
HER2 expression, *n* (%)^b^	
Breast cancer	
HER2 overexpressing	398 (34.6)
HER2 low expressing	110 (9.6)
Missing	2 (0.2)
Gastric cancer	
HER2 overexpressing	199 (17.3)
HER2 low expressing	44 (3.8)
Missing	51 (4.4)
Lung cancer	
IHC 3+	38 (3.3)
IHC 2+	89 (7.7)
IHC 1+	13 (1.1)
Missing	63 (5.5)
Colorectal cancer	
IHC 3+	43 (3.7)
IHC 2+	30 (2.6)
IHC 1+	15 (1.3)
Missing	19 (1.7)
Any HER2 mutation, *n* (%)	
Breast cancer	5 (0.4)
Gastric cancer	0
Lung cancer	75 (6.5)
Colorectal cancer	6 (0.5)
Age by tumor type, median (range), years^a^	
Breast cancer (*n* = 510)	56 (28-96)
Gastric cancer (*n* = 294)	65 (20-82)
Lung cancer (*n* = 203)	62 (23-88)
Colorectal cancer (*n* = 107)	59 (27-79)
Other cancer (*n* = 34)	58 (31-76)
Lung cancer or lung metastasis/lymphangitis carcinomatosis at baseline, *n* (%)	738 (64.2)
Lung comorbidities, *n* (%)^c^	81 (7.0)
Prior chest/lung radiotherapy, *n* (%)	190 (16.5)
Time since disease diagnosis, median (range), years^d^	2.98 (<0.1-30.9)
0 to ≤4 years, *n* (%)	624 (54.3)
>4 years, *n* (%)	403 (35.0)
No. of prior regimens, median (range)^d^	4.0 (1-27)
Lines of therapy in locally advanced/metastatic setting, *n* (%)^d^	
1-2	405 (35.2)
3-10	674 (58.6)
>10	40 (3.5)
Time from end date of last anticancer therapy to first infusion of T-DXd, median (range), months^d^	1.41 (0.3-54.5)
<Median (1.41 months), *n* (%)	518 (45.0)
≥Median (1.41 months), *n* (%)	531 (46.2)
T-DXd dose, *n* (%)	
5.4 mg/kg q3w	315 (27.4)
6.4 mg/kg q3w	808 (70.3)
>6.4 mg/kg q3w	27 (2.3)
Duration of treatment, median (range), months	5.81 (0.7-56.3)
0-6 months, *n* (%)	583 (50.7)
>6-12 months, *n* (%)	290 (25.2)
>12-24 months, *n* (%)	188 (16.3)
>24 months, *n* (%)	89 (7.7)
No. of treatment cycles, median (range)	8.0 (1-76)
WBC count, median (range), ×10^9^/l^d^	5.88 (0.9-77.5)
Weight, median (range), kg	59.70 (27.3-265.8)
Renal function, *n* (%)^e^	
Normal (serum creatinine clearance ≥90 ml/min)	470 (40.9)
Mild impairment (serum creatinine clearance ≥60 to <90 ml/min)	458 (39.8)
Moderate impairment (serum creatinine clearance ≥30 to <60 ml/min)	193 (16.8)
Severe impairment (serum creatinine clearance <30 ml/min)	3 (0.3)
Missing	26 (2.3)
Baseline albumin, median (range), g/l	39.0 (22-55)
Normal (≥3.5 g/dl), *n* (%)	937 (81.5)
Mild (≥3.0 to <3.5 g/dl), *n* (%)	151 (13.1)
Moderate (≥2.5 to <3.0 g/dl), *n* (%)	46 (4.0)
Severe (<2.5 g/dl), *n* (%)	3 (0.3)
Missing, *n* (%)	13 (1.1)
SpO_2_, *n* (%)	
≥95%	1080 (93.9)
<95%	57 (5.0)
Missing	13 (1.1)

ECOG PS, Eastern Cooperative Oncology Group performance status; HER2, human epidermal growth factor receptor 2; IHC, immunohistochemistry; q3w, every 3 weeks; SpO_2_, oxygen saturation; T-DXd, trastuzumab deruxtecan; WBC, white blood cell.

a Tumor type was missing for two patients.

b HER2 expression is evaluated using either central or local results according to the protocol defined enrollment criteria. Overexpressing was defined as IHC 3+ or *in situ* hybridization (ISH) positive for breast cancer, IHC 3+ or IHC 2+/ISH-positive for gastric cancer. Low expressing was defined as IHC 2+/ISH-negative or IHC 1+/not ISH-positive for breast cancer, or IHC 2+/ISH-negative or IHC 1+ for gastric cancer.

c Includes asthma, chronic obstructive pulmonary disease, prior interstitial lung disease/pneumonitis, pulmonary fibrosis, pulmonary emphysema, and radiation pneumonitis.

d Due to differences in data collection among the studies, some data were not collected for all patients; thus, the number of patients and percentages may not add up to 100% of the population.

e Renal function calculated based on creatinine clearance using the Cockcroft–Gault formula.

In all patients, the median treatment duration was 5.8 months (range, 0.7-56.3 months) (Table 2 and Supplementary Table S2, available at https://doi.org/10.1016/j.esmoop.2022.100554). Nearly half of the patients [49.3% (567/1150)] were treated for >6 months; 24.1% (277/1150) and 7.7% (89/1150) were treated for >12 and >24 months, respectively. Most patients treated for >12 months [69.0% (191/277)] had breast cancer.

### ILD/pneumonitis events and adjudication

A total of 276 potential ILD/pneumonitis events were sent for adjudication; 84.8% (234/276) were adjudicated as ILD/pneumonitis (including 224 drug-related and 10 non-drug-related events), 11.6% (32/276) were adjudicated as not being ILD/pneumonitis, and 3.6% (10/276) were pending adjudication at the data cut-off date.

**Figure 2:**
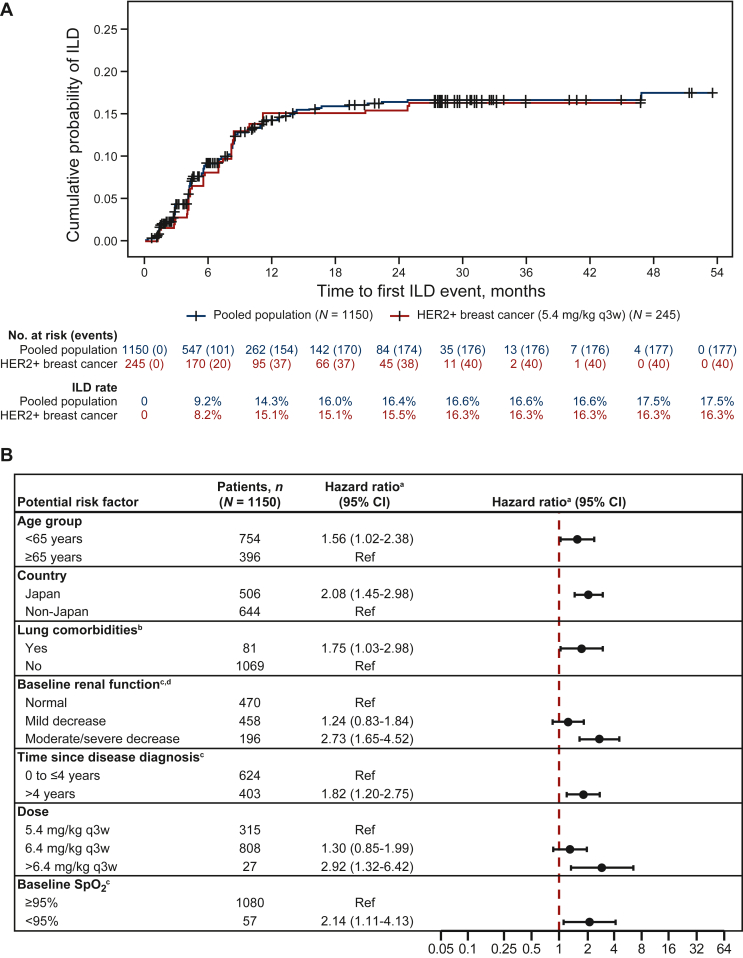
**Analysis of adjudicated drug-related ILD/pneumonitis events.** (A) Kaplan–Meier analysis of time to first adjudicated drug-related ILD/pneumonitis event. Among 177 patients who had ILD/pneumonitis, 154 (87.0%) had a first ILD/pneumonitis event within 12 months of starting treatment. The median time to adjudicated ILD/pneumonitis onset among those with ILD/pneumonitis was 5.4 months (range, <0.1-46.8 months). The median treatment duration in all patients in the pool was 5.8 months (range, 0.7-56.3 months), and 24.1% of all patients remained on treatment for >12 months. Treatment discontinuations due to reasons other than ILD/pneumonitis were included as a competing event. (B) Multivariate stepwise Cox regression analysis, final model. Factors included in the final model were age group, sex, country, Eastern Cooperative Oncology Group performance status, baseline weight, presence of lung cancer or lung metastasis/lymphangitis carcinomatosis at baseline, prior chest/lung radiotherapy, presence of lung comorbidity, baseline renal function, baseline white blood cell count, baseline albumin category, number of prior lines of therapy in the locally advanced/metastatic setting, time since disease diagnosis category, time since the end date of the last anticancer therapy to the first infusion of trastuzumab deruxtecan category, dose category, and baseline SpO_2_ category. Of these, seven factors were identified as factors of interest. HER2, human epidermal growth factor receptor 2; ILD, interstitial lung disease; q3w, every 3 weeks; Ref, reference; SpO_2_, oxygen saturation. ^a^Hazard ratios are presented relative to the reference categories indicated. ^b^Includes asthma, chronic obstructive pulmonary disease, prior interstitial lung disease/pneumonitis, pulmonary fibrosis, pulmonary emphysema, and radiation pneumonitis. ^c^Due to differences in data collection among the studies, some data were not collected for all patients; thus, the number of patients may not add up to the total population. ^d^Determined by Cockcroft–Gault formula.^37^

Overall, the AC identified drug-related ILD/pneumonitis in 177 of 1150 patients (15.4%) who had a total of 224 ILD/pneumonitis events. Most of these patients with ILD/pneumonitis [137/177 (77.4%); 11.9% (137/1150) of patients overall] had low-grade (worst grade of 1 or 2) ILD/pneumonitis; 15 patients (1.3%) had a grade 3 or 4 event, and 25 (2.2%) had a grade 5 event (Table 3). Overall, 87.0% (154/177) had a first adjudicated drug-related ILD/pneumonitis event within 12 months of starting T-DXd; 13.0% (23/177) had a first adjudicated drug-related ILD/pneumonitis event >12 months after starting T-DXd (Figure 2A).

**Table 3:** Adjudicated drug-related ILD/pneumonitis by tumor type and grade^a^

*n* (%)	Grade 1	Grade 2	Grade 3	Grade 4	Grade 5	Total
All patients (*N* = 1150)	48 (4.2)	89 (7.7)	14 (1.2)	1 (0.1)	25 (2.2)	177 (15.4)
Breast cancer (*n* = 510)	32 (6.3)	51 (10.0)	7 (1.4)	0	15 (2.9)	105 (20.6)
HER2-positive breast cancer treated with T-DXd 5.4 mg/kg q3w (*n* = 245)^b^	9 (3.7)	22 (9.0)	2 (0.8)	0	7 (2.9)	40 (16.3)
Gastric cancer (*n* = 294)	5 (1.7)	15 (5.1)	3 (1.0)	1 (0.3)	1 (0.3)	25 (8.5)
Lung cancer (*n* = 203)^c^	7 (3.4)	16 (7.9)	2 (1.0)	0	6 (3.0)	31 (15.3)
Colorectal cancer (*n* = 107)	0	5 (4.7)	1 (0.9)	0	3 (2.8)	9 (8.4)
Other cancer (*n* = 34)	4 (11.8)	2 (5.9)	1 (2.9)	0	0	7 (20.6)

HER2, human epidermal growth factor receptor 2; ILD, interstitial lung disease; q3w, every 3 weeks; T-DXd, trastuzumab deruxtecan.

a Patients with multiple ILD/pneumonitis events are listed only once in this table, based on the event with the highest grade.

b The HER2-positive breast cancer population (*n* = 245) is a subset of the entire breast cancer population (*n* = 510).

c All patients with lung cancer received 6.4 mg/kg q3w of T-DXd.

The median time to adjudicated drug-related ILD/pneumonitis onset was 5.4 months (range, <0.1-46.8 months). The median time to grade 5 onset was 3.2 months (range, <0.1-20.8 months). One patient with preexisting ILD/pneumonitis was adjudicated as having grade 5 ILD/pneumonitis. The ILD/pneumonitis onset date was assessed based on radiographic changes visualized by the AC and clinical features, when applicable. The AC identified ILD/pneumonitis onset earlier than investigators for 53.2% of events [99/186; median difference, 43 days (range, 1-499 days)] (Supplementary Table S3, available at https://doi.org/10.1016/j.esmoop.2022.100554). Among patients with earlier adjudicated onset according to the AC rather than the investigator, the distribution of investigator-assessed outcomes (based on the worst-grade event) appeared generally similar to the outcomes in patients for whom the investigator-reported and AC-assessed onset dates were the same (Supplementary Table S4, available at https://doi.org/10.1016/j.esmoop.2022.100554).

Among the 177 patients with AC-assessed drug-related ILD/pneumonitis, the first event was reported as grade 1 by the investigator in 42.9% of patients (76/177). Of these 76 patients with grade 1 ILD/pneumonitis, 47 were subsequently rechallenged with T-DXd as recommended (Table 1), and 3 of these 47 had a subsequent adjudicated (worst grade of 1) drug-related ILD/pneumonitis event (with onset ranging from 84 to 177 days after onset of the first adjudicated event).

### Cox multivariate regression analysis

Stepwise Cox regression evaluated whether there were associations between potential factors of interest and hazard of any-grade adjudicated drug-related ILD/pneumonitis. Seven baseline factors of interest were identified based on significance of association with the incidence of ILD/pneumonitis: age, enrollment in Japan, T-DXd dose, SpO_2_, moderate/severe renal impairment (based on the Cockcroft–Gault formula^37^), presence of lung comorbidities (not including lung cancer), and time since initial diagnosis (Figure 2B).

In the *post hoc* sensitivity analysis using the CKD-EPI formula^36^ rather than the Cockcroft–Gault formula, baseline renal function (as a categorical factor) was not found to be associated with the risk of adjudicated drug-related ILD/pneumonitis (Supplementary Figure S1, available at https://doi.org/10.1016/j.esmoop.2022.100554).

### Additional analyses of clinical importance

For 80.2% of grade 2-4 adjudicated drug-related ILD/pneumonitis events (85/106) and 84.6% of grade 5 events (22/26; 1 patient who died was listed with 2 events), one or more dose of corticosteroids was administered within 90 days of the adjudicated ILD/pneumonitis onset date. Due to the limitation of data collection, it is unknown how many of these patients received the optimal steroid treatment per the published ILD/pneumonitis management guidelines and study protocols.

The majority of patients (80.3%; 924/1150) did not receive prior immune checkpoint inhibitors (ICIs) (Supplementary Table S5, available at https://doi.org/10.1016/j.esmoop.2022.100554). The rate of any-grade adjudicated drug-related ILD/pneumonitis was 15.9% (147/924) in patients without prior ICI use [grade ≥3 in 3.6% (33/924)] and 13.3% (30/225) in patients with prior ICI use [grade ≥3 in 3.1% (7/225)]. Gastric cancer [*n* = 72/294 (24.5%)] and lung cancer [*n* = 135/203 (66.5%)] cohorts had the largest numbers of patients with prior ICI use, compared with <10 patients in each of the other cohorts. Adjudicated drug-related ILD/pneumonitis rates according to prior ICI use for each tumor type are summarized in Supplementary Table S5, available at https://doi.org/10.1016/j.esmoop.2022.100554.

## Discussion

T-DXd has shown antitumor activity in HER2-positive metastatic breast, gastric, and lung cancer; HER2-low metastatic breast cancer; HER2-mutant metastatic lung cancer; and other tumor types.^12^^,^^14^^,^^18^^,^^27^^,^^28^^,^^38^ This pooled analysis represents the most comprehensive evaluation of ILD/pneumonitis from the T-DXd clinical program, comprising 1150 heavily pretreated patients from nine phase I and II clinical trials. Overall, 177 patients (15.4%) experienced adjudicated drug-related ILD/pneumonitis, with most (77.4%) experiencing grade 1 or 2 events. In most patients (87.0%), the event occurred within 12 months of the first dose of T-DXd; median treatment duration was 5.8 months (range, 0.7-56.3 months). The longer T-DXd exposure in patients with breast cancer versus other tumor types may be due to these patients having longer progression-free survival and thus a longer treatment duration. It is notable that the median time to the first ILD/pneumonitis event was shorter for grade 5 events compared with the overall median time, suggesting that further investigation of this issue may be warranted. This represents the first data on T-DXd rechallenge following resolution of grade 1 ILD/pneumonitis, with only 3 of 47 rechallenged patients having recurrence of ILD/pneumonitis. Future research is warranted to better understand rechallenge with T-DXd.

The AC frequently identified ILD/pneumonitis onset as occurring earlier than did investigators, suggesting a need for additional ongoing vigilance. It is important to note, however, that the AC retrospectively reviewed patient records, with access to all clinical data and imaging scans during their assessment, whereas the investigator may not have had access to the same information during their assessment. While treating patients receiving T-DXd, health care providers should review prior CT scans for an assessment of ILD/pneumonitis along with their review of all medical records.

The rates of ILD/pneumonitis recently reported from the phase III randomized DESTINY-Breast03 trial of T-DXd as a second-line treatment (>70% of patients had received two or fewer prior lines of therapy in the metastatic setting) in patients with HER2-positive metastatic breast cancer were lower than those reported here.^13^ In DESTINY-Breast03, 10.5% of patients (27/257) treated with T-DXd experienced adjudicated drug-related ILD/pneumonitis, which was predominantly grade 1 or 2 [92.6% of patients (25/27)], with no grade 4 or 5 events.^13^ Increased knowledge of ILD/pneumonitis and implementation of ILD/pneumonitis monitoring, diagnosis, and management guidelines may explain why there were no grade 4 or 5 events since ILD/pneumonitis events may have been identified and treated early before they progressed.^23^ DESTINY-Breast03 investigated T-DXd as an earlier line of therapy than the trials included in this pooled analysis [median prior lines of therapy, 4 (range, 1-27)], which implies that perhaps there is a lower rate of ILD/pneumonitis in patients who are less heavily pretreated, although line of therapy was not identified as a potential factor of interest in the multivariate analysis reported here. The present analysis reflects a heavily pretreated population overall, including data from multiple tumor types, which may also partially explain differences in the rates of ILD/pneumonitis observed between the pooled analysis and DESTINY-Breast03. Several ongoing trials will continue to define the ILD/pneumonitis landscape in T-DXd.

A multivariate analysis was conducted to analyze factors potentially associated with ILD/pneumonitis. The analysis identified seven potential factors of interest with an increased hazard of drug-related ILD/pneumonitis; however, the clinical relevance of some of these factors remains unclear. Doses of T-DXd >6.4 mg/kg q3w had a significant association with increased ILD/pneumonitis hazard; for this reason, subsequent trials of T-DXd have treated patients at a dose of ≤6.4 mg/kg q3w.^18^^,^^39^^,^^40^

Most patients (70.3%) included in this pooled analysis received T-DXd 6.4 mg/kg q3w, which is the recommended dose approved for patients with certain types of gastric cancer and the dose being investigated in lung and colorectal cancer.^6^^,^^7^^,^^14^^,^^31^ Importantly, we did not find a significant difference in risk of ILD/pneumonitis between the 5.4 mg/kg q3w and the 6.4 mg/kg q3w doses in the multivariate Cox regression analysis. Thus, these data indicating a limited risk imposed by the 6.4 mg/kg q3w dose compared with the 5.4 mg/kg q3w dose of T-DXd provide valuable knowledge to clinicians regarding T-DXd-related ILD/pneumonitis when treating patients with multiple tumor types.

Baseline SpO_2_ <95% also posed a significant risk, but this was likely driven by a few patients with particularly low baseline SpO_2_. It is important to note that the 95% SpO_2_ threshold was chosen for practical (e.g. for sufficient patient numbers in each subset) rather than biological or clinical reasons, which precludes comprehensive assessment of the impact of SpO_2_.

Moderate or severe baseline renal impairment was also identified as a factor of interest in the preplanned multivariate analysis that used the Cockcroft–Gault formula; however, a subsequent *post hoc* analysis using the CKD-EPI formula showed a lack of association between severity of renal impairment (categorical) and ILD/pneumonitis risk. This analysis was completed because the CKD-EPI formula is Food and Drug Administration recommended for patients with renal impairment.^41^ The identification of renal function continues to be a clinical factor of interest for T-DXd-related ILD/pneumonitis and has been identified as a risk factor for ILD/pneumonitis related to other cancer therapies^23^; it is being explored in ongoing trials, including the randomized DESTINY-Breast03 trial.

The multivariate analysis suggested that younger patients (<65 years old) had a higher hazard of ILD/pneumonitis (after adjusting for other covariates selected by the model). This is not intuitive but may be due to unmeasured confounding factors among other clinical characteristics.

Patients treated with T-DXd in Japan had a higher hazard of ILD/pneumonitis compared with those treated outside of Japan. It is unknown whether this is due to biological factors or differences in monitoring and management practices in Japan; however, it is consistent with prior studies suggesting that other drug-induced lung injuries are more common in Japan than elsewhere.^20^^,^^23^^,^^33^^,^^42^

The presence of lung comorbidities was identified as a factor of interest in the multivariate model; however, lung cancer and lung metastases/lymphangitic carcinomatosis at baseline and prior chest/lung radiotherapy were not associated with ILD/pneumonitis in this analysis. While drug-related ILD/pneumonitis could be hypothesized to be related to a direct depot effect from binding to tumor deposits in the lung, the presence of ILD/pneumonitis in patients without lung involvement argues against this.

A *post hoc* analysis according to prior ICI use showed numerically similar rates of adjudicated drug-related ILD/pneumonitis in patients with or without prior ICI use.

### Limitations

In this analysis, the exact background rate of ILD/pneumonitis could not be determined because the AC did not review all patients; rather, a broad set of AE terms, including but not limited to ‘pneumonitis’ and ‘ILD,’ was used to trigger adjudication. Additionally, data suggest that steroid management was not optimal, with a delay in the detection of ILD/pneumonitis and dosing of steroids that was inconsistent with the recommendations in the ILD/pneumonitis treatment guidelines (e.g. many patients with grade ≥2 ILD/pneumonitis did not receive corticosteroids). Because steroid use (e.g. duration and route of administration) was inconsistently recorded in the clinical trial databases, we were not able to definitively distinguish cases in which steroids were administered in accordance with the current guidelines from those in which patients may have received an incomplete course of steroids or whose steroid treatment was unrelated to ILD/pneumonitis (e.g. topical steroids). The new guidelines for managing T-DXd-related ILD/pneumonitis that were implemented in December 2019^11^ were provided to health care providers and patients, along with specific education. However, many of the adjudicated ILD/pneumonitis events in these studies occurred before these guidelines were implemented.

The thresholds for the factors assessed by Cox multivariate regression were preselected based on medical judgment for practical reasons, which may impede the assessment of their impact on the risk of ILD/pneumonitis.

These analyses were also limited by the heterogeneity of the population (e.g. multiple tumor types, doses given) and duration of treatment. Due to this data heterogeneity, it is not possible to risk stratify patients. Nevertheless, the potential clinical factors of interest identified in these analyses are informative for clinicians on what to be aware of while treating patients with T-DXd. Future analyses of more homogeneous populations from randomized clinical trials will help confirm these findings and allow for further understanding of any relevant risk factors (e.g. specific to different tumor types).

### Conclusions

The overall incidence of adjudicated drug-related ILD/pneumonitis in T-DXd-treated patients was 15.4% across all doses and tumor types in this heavily pretreated population. Most cases were low grade, with a median time to onset of 5.4 months (range, <0.1-46.8 months). The median treatment duration was 5.8 months (range, 0.7-56.3 months). Risk-over-time assessments suggest that although ILD/pneumonitis development may require some cumulative exposure, a significant risk plateau was achieved after ∼12 months, and those who will develop this complication usually do so within 12 months of the first dose of T-DXd. This is based primarily on longer treatment duration in patients with breast cancer and suggests some individuals are inherently at lower risk of these events despite prolonged exposure. Longer T-DXd treatment duration and follow-up in patients with other tumor types are needed to confirm the observed trend. The monitoring, diagnosis, and management of ILD/pneumonitis is an area of continuing improvement; to this end, new toxicity guidelines have been implemented, and education has been provided to health care providers and patients. Patient awareness and ongoing education are critical elements to aid in early detection. In the DESTINY-Breast03 trial, lower rates of ILD/pneumonitis have been observed, with no patients experiencing a grade 4 or 5 event; these outcomes are likely due to a confluence of factors, including the less heavily pretreated study population and updated guidance for ILD/pneumonitis monitoring and management.^13^ Rechallenge with T-DXd after complete resolution of grade 1 events is possible and warrants further exploration; however, rechallenge is not recommended for all patients (e.g. patients with grade ≥2 ILD/pneumonitis). Potential clinical factors of interest for ILD/pneumonitis may include low SpO_2_, lung comorbidities, and renal impairment. Specific risk factors should be confirmed in ongoing and future trials. Phase III randomized controlled trials across multiple tumor types and in earlier lines of therapy are ongoing, and this analysis further supports the benefit–risk profile of T-DXd in advanced cancer.
