# Supplementary material for: Pooled analysis of drug-related interstitial lung disease and/or pneumonitis in nine trastuzumab deruxtecan monotherapy studies
Source: ESMO Open. 2022 Aug 11;7(4):100554. doi: 10.1016/j.esmoop.2022.100554 (PMC9434416; doi:10.1016/j.esmoop.2022.100554)
Supplement: Supplementary Material [file mmc1.docx]

Supplementary Material

Pooled Analysis of Drug-Related Interstitial Lung Disease and/or Pneumonitis in 9 Trastuzumab Deruxtecan Monotherapy Studies

Charles A. Powell, MD, MBA^a^; Shanu Modi, MD^b^; Hiroji Iwata, PhD^c^; Shunji Takahashi, MD, PhD^d^; Egbert F. Smit, MD, PhD^e^; Salvatore Siena, MD^f,g^; Dwan-Ying Chang, MD^h^; Euan Macpherson, MSc^i^; Amy Qin, PhD^j^; Jasmeet Singh, MD, MPHA^j^; Corina Taitt, MD^j^; Norah Shire, PhD, MPH^i^; D. Ross Camidge, MD, PhD^k^

^a^Catherine and Henry J. Gaisman Division of Pulmonary Critical Care and Sleep Medicine, Icahn School of Medicine at Mount Sinai, 1 Gustave L. Levy Place, New York, NY 10029, USA (charles.powell@mssm.edu); ^b^Memorial Sloan Kettering Cancer Center, 1275 York Ave, New York, NY 10065, USA (modis@mskcc.org); ^c^Aichi Cancer Center Hospital, 1-1 Kanokoden, Chikusa Ward, Nagoya, Aichi 464-0021, Japan (hiwata@aichi-cc.jp); ^d^Medical Oncology, The Cancer Institute Hospital of JFCR, 3-8-31, Ariake, Koto, Tokyo 135-8550, Japan (s.takahashi-chemotherapy@jfcr.or.jp); ^e^Department of Thoracic Oncology, Netherlands Cancer Institute, Plesmanlaan 121, 1066 CX Amsterdam, the Netherlands (e.smit@nki.nl); ^f^Department of Oncology and Hemato-Oncology, Università degli Studi di Milano, Via Festa del Perdono, 7, 20122 Milano MI, Italy (salvatore.siena@unimi.it); ^g^Niguarda Cancer Center, Grande Ospedale Metropolitano Niguarda, Piazza dell'Ospedale Maggiore, 3, 20162 Milano MI, Italy; ^h^National Taiwan University Hospital, No. 7號, Zhongshan S Rd, Zhongzheng District, Taipei City 100, Taiwan (dwanying@gmail.com); ^i^AstraZeneca Pharmaceuticals, 1 Medimmune Way, Gaithersburg, MD 20878, USA (EM: euan.macpherson@astrazeneca.com; NS:norah.shire@astrazeneca.com); ^j^Daiichi Sankyo Inc., 211 Mt Airy Rd, Basking Ridge, NJ 07920, USA (AQ: aqin@dsi.com; JS: jsingh@dsi.com; CT: ctaitt@dsi.com); ^k^University of Colorado Cancer Center, 1665 Aurora Court Anschutz Cancer Pavilion, Aurora, CO 80045, USA (ross.camidge@cuanschutz.edu)

# Adjudication Committee (AC)

The interstitial lung disease (ILD)/pneumonitis AC was formed jointly by Daiichi Sankyo and AstraZeneca to ensure that all ILD/pneumonitis events identified are judged in a consistent, uniform manner, using standard criteria. The AC is composed of medical oncologists, pulmonologists, and radiologists with representation from the US, Europe, and Japan/Asia.

The AC reviews all potential ILD/pneumonitis cases, defined as any adverse event (AE) reported under the following terms:

The current list of Medical Dictionary for Regulatory Activities Preferred Terms includes “acute interstitial pneumonitis,” “acute respiratory distress syndrome,” “acute respiratory failure,” “allergic eosinophilia,” “alveolar lung disease,” alveolar proteinosis,” “alveolitis,” “alveolitis allergic,” “alveolitis necrotizing,” “autoimmune lung disease,” “bronchiolitis,” “bronchiolitis obliterans syndrome,” “combined pulmonary fibrosis and emphysema,” “diffuse alveolar damage,” “eosinophilia myalgia syndrome,” “eosinophilic granulomatosis with polyangiitis,” “eosinophilic pneumonia,” “eosinophilic pneumonia acute,” “eosinophilic pneumonia chronic,” “granulomatous pneumonitis,” “hypersensitivity pneumonitis,” “idiopathic interstitial pneumonia,” “idiopathic pneumonia syndrome,” “idiopathic pulmonary fibrosis,” “immune-mediated pneumonitis,” “interstitial lung disease,” “lung infiltration,” “lung opacity,” “necrotizing bronchiolitis,” “obliterative bronchiolitis,” “organizing pneumonia,” “pleuroparenchymal fibroelastosis,” “pneumonitis,” “progressive massive fibrosis,” “pulmonary fibrosis,” “pulmonary necrosis,” “pulmonary radiation injury,” “pulmonary sarcoidosis,” “pulmonary toxicity,” “pulmonary vasculitis,” “radiation alveolitis,” “radiation fibrosis–lung,” “radiation pneumonitis,” “respiratory failure,” “restrictive pulmonary disease,” “rheumatoid lung,” “sarcoidosis,” “small airways disease,” and “transfusion-related acute lung injury.”

This list of terms will be updated periodically, and additional AEs outside of the predefined list of terms can also be sent for adjudication

# Grading of Adjudicated ILD/Pneumonitis Events

Events adjudicated as ILD/pneumonitis are graded according to the criteria outlined in the National Cancer Institute Common Terminology Criteria for Adverse Events version 5.0^1^ for AEs of “pneumonitis” as follows:

- Grade 1: asymptomatic; clinical or diagnostic observations only; intervention not indicated
- Grade 2: symptomatic; medical intervention indicated; limited instrumental activities of daily living
- Grade 3: severe symptoms; limited self-care activities of daily living; oxygen indicated
- Grade 4: life-threatening respiratory compromise: urgent intervention indicated (eg, tracheotomy or intubation)
- Grade 5: death

For this analysis, in cases where steroids were administered for asymptomatic events, the AC considered the ILD/pneumonitis event to be grade 2 if it was adjudicated prior to the guidelines modification, whereas the current guidelines for management of trastuzumab deruxtecan (T-DXd)–related ILD/pneumonitis consider all asymptomatic events to be grade 1 (regardless of steroid use)^2^; thus, some of the events considered as grade 2 in this analysis might be considered as grade 1 according to the current guidelines.

# Data Used for Assessment of ILD/pneumonitis by the AC

The following supporting documents were provided to the AC (if available) for all potential ILD/pneumonitis events:

- ILD/pneumonitis checklist completed by the site
- Admission summary/outpatient clinical summary with history/physical exam, presenting signs and symptoms of ILD/pneumonitis and testing performed
- Discharge summary or clinical summary (if not hospitalized) containing treatment prescribed and outcome of event
- Pulmonary specialist consult containing the patient evaluation and findings of the pulmonary specialist if obtained
- Pulmonary function tests including diffusing capacity of the lungs for carbon monoxide, pulse oximetry (oxygen saturation [SpO2]) at baseline (during screening period), right before ILD/pneumonitis event onset, during the event, and after resolution, if available
- Relevant laboratory reports with reference ranges (baseline, just prior to event date, during event, and after resolution if available)
- COVID-19 testing (if available)
- Serum markers testing (eg, KL-6, SP-D, or others as applicable)
- Molecular testing: results of HER2, EGFR, ALK, ROS, K-RAS, as applicable
- Cultures: sputum, urine, blood during the event as applicable
- Pathology reports: bronchoalveolar lavage and transbronchial lung biopsy as applicable
- Radiology images: baseline computed tomography (CT) scan (prior to dosing), CT/chest x-ray obtained just prior to event date, CT (preferably high-resolution CT) obtained for diagnosis, follow up CT/chest x-rays (progression/resolution), and/or chest magnetic resonance imaging/positron emission tomography scans (as applicable)
- Council for Investigational Organizations of Medical Sciences report
- Autopsy report as applicable
- Death certificate as applicable
- Witness description of death if other reports not available
- Signed physician narrative, mandatory in absence of any recommended documents (must include a concise narrative by the primary investigator including description of the event, treatment, and outcome, as well as why the documents were not available, except where a note to file has been provided)
- Electronic case report form profile report

# Cox Multivariate Stepwise Regression Analysis

This analysis was based on standard Cox regression (without accounting for competing events such as withdrawal from treatment prior to an ILD/pneumonitis event). Potential risk factors included for selection were age group (<65 vs ≥65 years), sex, country (Japan vs non-Japan), Eastern Cooperative Oncology Group performance status (0 vs >0), baseline weight, presence of lung cancer or lung metastases/lymphangitis carcinomatosis at baseline, prior chest/lung radiotherapy, presence of lung comorbidity (defined as asthma, chronic obstructive pulmonary disease, prior ILD/pneumonitis, pulmonary fibrosis, pulmonary emphysema, or radiation pneumonitis), baseline renal function category (using the Cockcroft-Gault formula for creatinine clearance), baseline white blood cell count, baseline albumin category (normal vs mild decrease vs moderate/severe decrease), number of prior lines of therapy in the locally advanced/metastatic setting category (1-2 vs 3-10 vs >10), time since disease diagnosis category (≤4 vs >4 years), time from end date of last anticancer therapy to first infusion of T-DXd category (< median vs ≥ median), dose category (5.4 vs 6.4 vs >6.4 mg/kg every 3 weeks), and baseline oxygen saturation category (≥95% vs <95%).

# Renal Function Impairment Post Hoc Analysis

Given the identification of renal impairment as a potential factor of interest, a further post hoc senstivity analysis was conducted using the Chronic Kidney Disease Epidemiology Collaboration (CKD-EPI) formula rather than the Cockcroft-Gault formula to evaluate renal function.^3^ Compared with the Cockcroft-Gault formula, the CKD-EPI formula categorized fewer patients as having moderate/severe renal impairment and more patients as having mild renal impairment or normal renal function (**Table S6**). In a multivariate stepwise Cox regression analysis using CKD-EPI as a categorical factor, baseline renal function was not found to be associated with risk of adjudicated drug-related ILD/pneumonitis (**Figure S1**). The identification of renal function as a factor of interest in the preplanned analysis remains unexplained. It is not believed to be due to accumulation of T-DXd or released DXd in patients’ bodies as T-DXd is primarily eliminated through the liver (in cynomolgus monkeys, 18.7% of T-DXd was excreted in urine vs 67.3% in feces within 336 hours [14 days]).^4^ Although the Cockcroft-Gault formula is widely used, it has been suggested that the newer CKD-EPI formula provides a better estimate of renal function.^3,5-7^ Details of the inclusion criteria from the trials included in this analysis are provided to more completely describe the patient population enrolled (**Table S7**).

# Creatinine Clearance Calculations

The Cockcroft-Gault formula is widely used to estimate creatinine clearance. Estimates of creatinine clearance are based on serum creatinine, sex, age, and weight.^6^ Accuracy of the Cockcroft-Gault formula is impacted by weight and body mass index in addition to age.^5^

The CKD-EPI formula is a more recently developed formula used to calculate glomerular filtration rate based on serum creatinine, sex, age, and race.^7^ It has been suggested that the CKD-EPI formula provides a better estimate of renal function. Accuracy of the CKD-EPI formula is impacted by patients’ age and glomerular filtration rate.^5^ The CKD-EPI formula is now the recommended formula for estimating glomerular filtration rate according to the National Kidney Foundation’s Kidney Disease Outcomes Quality Initiative.^3^

# Table S1. Summary of Studies Included in the Analysis

| Study identifier | Study design | Number of patients | T-DXd dose | Indications |
| --- | --- | --- | --- | --- |
| DS8201-A-J101 (NCT02564900)^8,9^ | Phase 1, 2-part, multicenter, nonrandomized, open-label, multiple-dose, first-in-human study | 280 | 0.8-8.0 mg/kg q3w | Advanced solid malignant tumors, including advanced breast cancer or gastric/GEJ adenocarcinoma, and  other HER2-expressing solid tumors |
| DS8201-A-J102 (NCT03366428)^10^ | Phase 1, multicenter, nonrandomized, open-label, multiple-dose study | 51 | 6.4 mg/kg q3w | HER2-expressing metastatic and/or unresectable breast cancer |
| DS8201-A-A104 (NCT03383692)^10^ | Phase 1, multicenter, nonrandomized, open-label, single-sequence crossover DDI study | 40 | 5.4 mg/kg q3w | HER2-expressing advanced solid tumors |
| DS8201-A-A103 (NCT03368196)^10^ | Phase 1, multicenter, nonrandomized, open-label study | 12 | 6.4 mg/kg q3w | HER2-positive advanced unresectable and/or refractory gastric/GEJ adenocarcinoma or breast cancer |
| DESTINY-Breast01 (NCT03248492; DS8201-A-U201)^2^ | Phase 2, 2-part, global, multicenter, randomized, open-label, multiple-dose study | 253 | 5.4, 6.4, or 7.4 mg/kg q3w | T-DM1–treated HER2-positive unresectable and/or metastatic breast cancer |
| DESTINY-Gastric01 (NCT03329690; DS8201-A-J202)^11^ | Phase 2, multicenter, randomized, open-label study | 170 | 6.4 mg/kg q3w | HER2-positive advanced gastric/GEJ adenocarcinoma |
| DESTINY-CRC01 (NCT03384940; DS8201-A-J203)^12^ | Phase 2, multicenter, global, nonrandomized, open-label study | 86 | 6.4 mg/kg q3w | HER2-positive advanced CRC |
| DESTINY-Lung01 (NCT03505710; DS8201-A-U204)^13^ | Phase 2, multicenter, global, nonrandomized, open-label study | 179 | 6.4 mg/kg q3w | HER2-overexpressing or ‑mutation–positive unresectable and/or metastatic NSCLC |
| DESTINY-Gastric02 (NCT04014075; DS8201-A-U205)^14^ | Phase 2, multicenter, global, nonrandomized, open-label study | 79 | 6.4 mg/kg q3w | HER2-positive unresectable or metastatic gastric/GEJ adenocarcinoma |

CRC, colorectal cancer; DDI, drug-drug interaction; GEJ, gastroesophageal junction; HER2, human epidermal growth factor receptor 2; NSCLC, non-small cell lung cancer; q3w, every 3 weeks; T-DM1, trastuzumab emtansine; T-DXd, trastuzumab deruxtecan.

# Table S2. Summary of T-DXd Exposure Time

|  | Duration of treatment, median (min, max), mo | ≤12 mo treatment duration, n (%) | >12 to ≤24 mo treatment duration, n (%) | >24 mo treatment duration, n (%) |
| --- | --- | --- | --- | --- |
| All patients (N=1150) | 5.8 (0.7, 56.3) | 873 (75.9) | 188 (16.3) | 89 (7.7) |
| Breast cancer (n=510) | 8.7 (0.7, 56.3) | 319 (62.5) | 113 (22.2) | 78 (15.3) |
| HER2-positive breast cancer treated with 5.4 mg/kg q3w (n=245)^a^ | 9.7 (0.7, 46.9) | 141 (57.6) | 57 (23.3) | 47 (19.2) |
| Gastric cancer (n=294) | 4.1 (0.7, 53.2) | 264 (89.8) | 24 (8.2) | 6 (2.0) |
| Lung cancer (n=203) | 4.8 (0.7, 22.5) | 172 (84.7) | 31 (15.3) | 0 |
| Colorectal cancer (n=107) | 3.0 (0.7, 22.3) | 95 (88.8) | 12 (11.2) | 0 |
| Other cancer (n=34) | 8.5 (0.7, 51.8) | 21 (61.8) | 8 (23.5) | 5 (14.7) |

HER2, human epidermal growth factor receptor 2; q3w, every 3 weeks; T-DXd, trastuzumab deruxtecan.

^a^ The HER2-positive breast cancer population (n=245) is a subset of the entire breast cancer population (n=510).

# Table S3. Investigator-Assessed ILD/Pneumonitis Onset Date vs Adjudication Committee-Assessed ILD/Pneumonitis Onset Date

|  | Adjudicated drug-related ILD/pneumonitis events  (n=186) |
| --- | --- |
| **Concordant, n (%)** | **80 (43.0)** |
| **Discordant** |  |
| **Earlier** than the adjudication committee, n (%) | 7 (3.8) |
| **Median difference (range), days** | 4.0 (1-44) |
| **Later** than the adjudication committee, n (%) | 99 (53.2) |
| **Median difference (range), days** | 43.0 (1-499) |

ILD, interstitial lung disease.

# Table S4. Summary of ILD/Pneumonitis Outcomes by Investigator-Assessed ILD/Pneumonitis Onset Date vs Adjudication Committee-Assessed ILD/Pneumonitis Onset Date

|  | Outcome of the worst grade ILD/pneumonitis event | | | | | |
| --- | --- | --- | --- | --- | --- | --- |
|  | **Fatal** | **Not recovered/ not resolved** | **Recovering/ resolving** | **Recovered/ resolved with sequelae** | **Recovered/ resolved** | **Missing/ unknown** |
| **Concordant, n (%) (n=75)^a^** | 4 (5.3) | 40 (53.3) | 7 (9.3) | 4 (5.3) | 20 (26.7) | — |
| **Discordant** |  | | | | | |
| **Earlier** than the adjudication committee, n (%) (n=7)^a^ | 1 (14.3) | 4 (57.1) | — | — | 2 (28.6) | — |
| **Later** than the adjudication committee, n (%) (n=97)^a^ | 6 (6.2) | 39 (40.2) | 8 (8.2) | 7 (7.2) | 35 (36.1) | 2 (2.1) |

ILD, interstitial lung disease.

^a^ Number of patients who had ≥1 adjudicated ILD/pneumonitis event in the category. Some patients may have had >1 event.

# Table S5. Adjudicated Drug-Related ILD/Pneumonitis Events by Tumor Type and Prior ICI Use^a,b^

|  | Grade 1 | Grade 2 | Grade 3 | Grade 4 | Grade 5 | Total |
| --- | --- | --- | --- | --- | --- | --- |
| Patients with prior ICI use, n (%) | | | | | | |
| All patients (n=225) | 6 (2.7) | 17 (7.6) | 0 | 1 (0.4) | 6 (2.7) | **30 (13.3)** |
| Breast cancer (n=9) | 0 | 1 (11.1) | 0 | 0 | 0 | **1 (11.1)** |
| HER2-positive breast cancer treated with 5.4 mg/kg q3w (n=5)^c^ | 0 | 1 (20.0) | 0 | 0 | 0 | **1 (20.0)** |
| Gastric cancer (n=72) | 1 (1.4) | 4 (5.6) | 0 | 1 (1.4) | 0 | **6 (8.3)** |
| Lung cancer (n=135) | 4 (3.0) | 12 (8.9) | 0 | 0 | 6 (4.4) | **22 (16.3)** |
| Colorectal cancer (n=4) | 0 | 0 | 0 | 0 | 0 | **0** |
| Other cancer (n=5) | 1 (20.0) | 0 | 0 | 0 | 0 | **1 (20.0)** |
| Patients with no prior ICI use, n (%) | | | | | | |
| All patients (n=924) | 42 (4.5) | 72 (7.8) | 14 (1.5) | 0 | 19 (2.1) | **147 (15.9)** |
| Breast cancer (n=501) | 32 (6.4) | 50 (10.0) | 7 (1.4) | 0 | 15 (3.0) | **104 (20.8)** |
| HER2-positive breast cancer treated with 5.4 mg/kg q3w (n=240)^c^ | 9 (3.8) | 21 (8.8) | 2 (0.8) | 0 | 7 (2.9) | **39 (16.3)** |
| Gastric cancer (n=67) | 3 (4.5) | 4 (6.0) | 2 (3.0) | 0 | 0 | **9 (13.4)** |
| Lung cancer (n=103) | 0 | 5 (4.9) | 1 (1.0) | 0 | 3 (2.9) | **9 (8.7)** |
| Colorectal cancer (n=222) | 4 (1.8) | 11 (5.0) | 3 (1.4) | 0 | 1 (0.5) | **19 (8.6)** |
| Other cancer (n=29) | 3 (10.3) | 2 (6.9) | 1 (3.4) | 0 | 0 | **6 (20.7)** |

HER2, human epidermal growth factor receptor 2; ICI, immune checkpoint inhibitor; ILD, interstitial lung disease; q3w, every 3 weeks.

^a^ Patients with multiple ILD/pneumonitis events are listed only once in this table, based on the event with the highest grade.

^b^ One patient provided no information about prior or concomitant medication and was excluded from this analysis.

^c^ The HER2-positive breast cancer population is a subset of the entire breast cancer population.

# Table S6. Comparison of Renal Function Categories Using the Individualized CKD-EPI Formula vs the CG Formula

| Renal function categories based on CrCl (CG formula), (mL/min) | Renal function based on eGFR using individualized CKD‑EPI formula, (mL/min) | | | | |
| --- | --- | --- | --- | --- | --- |
|  | **Normal** | **Mild** | **Moderate/ severe** | **Missing** | **Total** |
| **Normal** | 439 | 25 | 0 | 6 | 470 |
| **Mild** | 79 | 371 | 2 | 6 | 458 |
| **Moderate/severe** | 0 | 103 | 91 | 2 | 196 |
| **Missing** | 11 | 5 | 4 | 6 | 26 |
| **Total** | 529 | 504 | 97 | 20 | 1150 |

CG, Cockcroft-Gault; CKD-EPI, Chronic Kidney Disease Epidemiology Collaboration; CrCl, creatinine clearance; eGFR, estimated glomerular filtration rate.

# Table S7. Summary of Protocol-Specified Renal Function Inclusion Criteria for the Studies Included in the Analysis

| Study identifier | Indications | Renal function criteria |
| --- | --- | --- |
| DS8201-A-J101 (NCT02564900)^8,9^ | Advanced solid malignant tumors, including advanced breast cancer or gastric/GEJ adenocarcinoma, and other HER2-expressing solid tumors | Adequate renal function within 7 days before registration, defined as creatinine clearance ≥60 mL/min as calculated using the modified Cockcroft-Gault equation^6^ or serum creatinine ≤1.5×ULN |
| DS8201-A-J102 (NCT03366428) | HER2-expressing metastatic and/or unresectable breast cancer | Adequate renal function, defined as creatinine clearance ≥30 mL/min as calculated using the Cockcroft-Gault equation^6^ |
| DS8201-A-A104 (NCT03383692) | HER2-expressing advanced solid tumors | Adequate renal function within 14 days before enrollment, defined as creatinine clearance ≥30 mL/min as calculated using the Cockcroft-Gault equation^6^ |
| DS8201-A-A103 (NCT03368196) | HER2-positive advanced unresectable and/or refractory gastric/GEJ adenocarcinoma or breast cancer | Adequate renal function within 7 days before registration, defined as creatinine clearance ≥60 mL/min as calculated using the Cockcroft-Gault equation^6^ or serum creatinine ≤1.5×ULN |
| DESTINY-Breast01 (NCT03248492; DS8201-A-U201)^2^ | T-DM1–treated, HER2-positive unresectable and/or metastatic breast cancer | Adequate renal function, defined as creatinine clearance ≥30 mL/min as calculated using the Cockcroft-Gault equation^6^ |
| DESTINY-Gastric01 (NCT03329690; DS8201-A-J202)^11^ | HER2-positive advanced gastric/GEJ adenocarcinoma | Adequate renal function within 14 days before registration, defined as creatinine clearance ≥30 mL/min as calculated using the Cockcroft-Gault equation^6^ |
| DESTINY-CRC01 (NCT03384940; DS8201-A-J203)^12^ | HER2-positive advanced CRC | Adequate renal function within 14 days before enrollment, defined as creatinine clearance ≥30 mL/min as calculated using the Cockcroft-Gault equation^6^ |
| DESTINY-Lung01 (NCT03505710; DS8201-A-U204)^13^ | HER2-overexpressing or ‑positive unresectable and/or metastatic NSCLC | Adequate renal function within 14 days before registration, defined as creatinine clearance ≥30 mL/min as calculated using the Cockcroft-Gault equation^6^ |
| DESTINY-Gastric02 (NCT04014075; DS8201-A-U205) | HER2-positive unresectable or metastatic gastric/GEJ adenocarcinoma | Adequate renal function within 14 days before enrollment, defined as creatinine clearance ≥30 mL/min as calculated using the Cockcroft-Gault equation^6^ |

CRC, colorectal cancer; GEJ, gastroesophageal junction; HER2, human epidermal growth factor receptor 2; NSCLC, non-small cell lung cancer; ULN, upper limit of normal.

# Figure S1. Sensitivity Analysis Using the CKD-EPI Individualized Formula for Renal Function as a Categorical Variable Within a Multivariate Stepwise Cox Regression, Final Model^a^


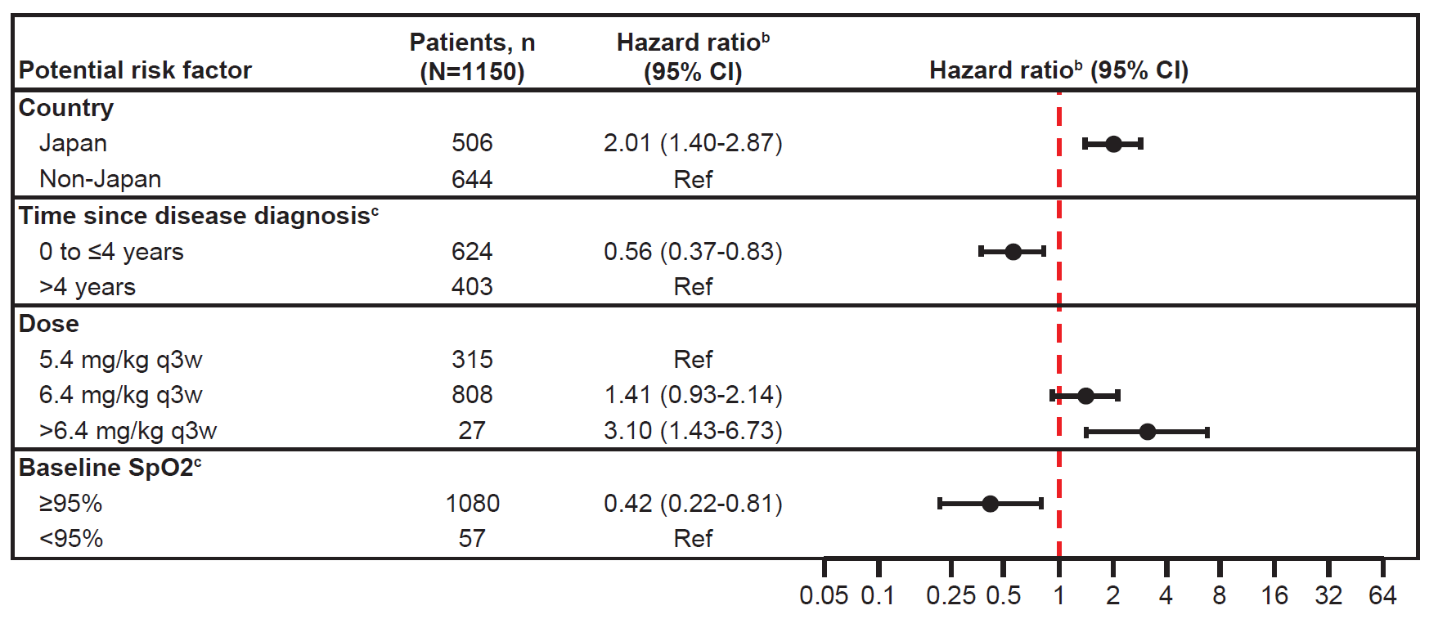


CKD-EPI, Chronic Kidney Disease Epidemiology Collaboration; q3w, every 3 weeks; Ref, reference; SpO2, oxygen saturation.

^a^ Factors included in the model were age group, sex, country, Eastern Cooperative Oncology Group performance status, baseline weight, presence of lung cancer or lung metastasis/lymphangitis carcinomatosis at baseline, prior chest/lung radiotherapy, presence of lung comorbidity, baseline CKD-EPI estimated glomerular filtration rate (individual body surface area; mL/min categorized), baseline white blood cell count, baseline albumin category, number of prior lines of therapy in the locally advanced/metastatic setting, time since disease diagnosis category, time since end date of last anticancer therapy to first infusion of trastuzumab deruxtecan category, dose category, and baseline SpO2 category. Of these, 4 factors were identified as factors of interest.

^b^ Hazard ratios are presented relative to the reference categories indicated.

^c^ Due to differences in data collection among the studies, some data were not collected for all patients; thus, the number of patients may not add up to the total population.

# References

1. National Cancer Institute Common Terminology Criteria for Adverse Events (CTCAE) version 5.0. Accessed July 27, 2021. <https://ctep.cancer.gov/protocoldevelopment/electronic_applications/docs/ctcae_v5_quick_reference_5x7.pdf>

2. Modi S, Saura C, Yamashita T, et al. Trastuzumab deruxtecan in previously treated HER2-positive breast cancer. *N Engl J Med*. 2020;382(7):610–621. <https://doi.org/10.1056/NEJMoa1914510>

3. Vassalotti JA, Centor R, Turner BJ, Greer RC, Choi M, Sequist TD. Practical approach to detection and management of chronic kidney disease for the primary care clinician. *Am J Med*. 2016;129(2):153–162.e7. <https://doi.org/10.1016/j.amjmed.2015.08.025>

4. Nagai Y, Oitate M, Shiozawa H, Ando O. Comprehensive preclinical pharmacokinetic evaluations of trastuzumab deruxtecan (DS-8201a), a HER2-targeting antibody-drug conjugate, in cynomolgus monkeys. *Xenobiotica*. Sep 2019;49(9):1086–1096. <https://doi.org/10.1080/00498254.2018.1531158>

5. Michels WM, Grootendorst DC, Verduijn M, Elliott EG, Dekker FW, Krediet RT. Performance of the Cockcroft-Gault, MDRD, and new CKD-EPI formulas in relation to GFR, age, and body size. *Clin J Am Soc Nephrol*. 2010;5(6):1003–1009. <https://doi.org/10.2215/cjn.06870909>

6. Cockcroft DW, Gault MH. Prediction of creatinine clearance from serum creatinine. *Nephron*. 1976;16(1):31–41. <https://doi.org/10.1159/000180580>

7. Levey AS, Stevens LA, Schmid CH, et al. A new equation to estimate glomerular filtration rate. *Ann Intern Med*. May 5 2009;150(9):604–612. <https://doi.org/10.7326/0003-4819-150-9-200905050-00006>

8. Tamura K, Tsurutani J, Takahashi S, et al. Trastuzumab deruxtecan (DS-8201a) in patients with advanced HER2-positive breast cancer previously treated with trastuzumab emtansine: a dose-expansion, phase 1 study. *Lancet Oncol*. 2019;20(6):816–826. <https://doi.org/10.1016/S1470-2045(19)30097-X>

9. Doi T, Shitara K, Naito Y, et al. Safety, pharmacokinetics, and antitumour activity of trastuzumab deruxtecan (DS-8201), a HER2-targeting antibody-drug conjugate, in patients with advanced breast and gastric or gastro-oesophageal tumours: a phase 1 dose-escalation study. *Lancet Oncol*. 2017;18(11):1512–1522. <https://doi.org/10.1016/S1470-2045(17)30604-6>

10. Center for Drug Evaluation and Research. Multi-discipline review: Enhertu. Accessed April 29, 2021. <https://www.accessdata.fda.gov/drugsatfda_docs/nda/2019/761139Orig1s000MultidisciplineR.pdf>

11. Shitara K, Bang YJ, Iwasa S, et al. Trastuzumab deruxtecan in previously treated HER2-positive gastric cancer. *N Engl J Med*. 2020;382(25):2419–2430. <https://doi.org/10.1056/NEJMoa2004413>

12. Siena S, Bartolomeo MD, Raghav KPS, et al. A phase II, multicenter, open-label study of trastuzumab deruxtecan (T-DXd; DS-8201) in patients (pts) with HER2-expressing metastatic colorectal cancer (mCRC): DESTINY-CRC01. Presented at: American Society of Clinical Oncology Annual Meeting 2020; May 29–31, 2020; virtual. Abstract 4000.

13. Li BT, Smit EF, Goto Y, et al. Trastuzumab deruxtecan in HER2-mutant non–small-cell lung cancer. *N Engl J Med*. 2022;386(3):241–251. <https://doi.org/10.1056/NEJMoa2112431>

14. Kotani D, Shitara K. Trastuzumab deruxtecan for the treatment of patients with HER2-positive gastric cancer. *Ther Adv in Med Oncol*. 2021;13:1758835920986518. <https://doi.org/10.1177/1758835920986518>
